# Correlation between red blood cell distribution width and deep vein thrombosis in patients with lower limb fractures

**DOI:** 10.3389/fcvm.2026.1841161

**Published:** 2026-07-31

**Authors:** Shuai Ji, Hongfei Qi, Zhong Li, Bo Wu, Bing Du, Xianjie Ai, Chengcheng Zhang, Kun Zhang, Yanling Yang, Ming Li

**Affiliations:** 1 Department of Traumatic Orthopedics, Honghui Hospital, Xi’an Jiaotong University, Xi’an, Shaanxi, China; 2 Medical College of Yan'an University, Yan'an, Shaanxi, China

**Keywords:** DVT, logistic regression, lower limb fracture, red cell distribution width, risk factor

## Abstract

**Objective:**

This study aimed to investigate the relationship between red blood cell distribution width (RDW) and perioperative lower-extremity deep vein thrombosis (DVT) in patients aged 18–75 years with lower-limb fractures.

**Material and methods:**

From February 2021 to February 2023, we included 766 patients aged 18–75 years with lower-limb fractures. Univariate and multivariable analyses were used to investigate the association between RDW and DVT. The discriminatory ability and cohort-specific exploratory cut-off values of RDW were evaluated using receiver operating characteristic (ROC) curves.

**Results:**

ROC curve analysis identified cohort-specific exploratory cut-off values of 43.9 fL for RDW standard deviation (RDW-SD) and 13.2% for RDW coefficient of variation (RDW-CV), and RDW-SD showed better discrimination than RDW-CV (*P* < 0.05). In the adjusted analyses, RDW-SD ≥43.9 fL [adjusted odds ratio (aOR), 3.372; *P* < 0.001], RDW-CV ≥13.2% (aOR, 2.556; *P* < 0.001), advanced age (aOR, 1.054; *P* < 0.001), higher platelet count (PLT; per 10 × 10⁹/L increase: aOR, 1.115; *P* = 0.010), and Hb <120 g/L (aOR, 1.785; *P* < 0.001) were independently associated with perioperative DVT. Significant differences in age, fracture site, DVT, diabetes, cancer, and Hb <120 g/L were observed between the RDW-SD ≥43.9 fL and RDW-SD <43.9 fL groups (all *P* < 0.05).

**Conclusion:**

Advanced age, Hb <120 g/L, higher PLT, RDW-SD ≥43.9 fL, and RDW-CV ≥13.2% were independently associated with perioperative DVT in patients aged 18–75 years with lower-limb fractures. RDW-SD showed better discrimination than RDW-CV.

## Introduction

Deep vein thrombosis (DVT) is a common and serious perioperative complication of lower extremity fractures, especially during trauma and surgical interventions, and its incidence is significantly increased, which may lead to pulmonary embolism (PE), long-term dysfunction, and even death. In orthopaedic surgery, patients with lower limb fractures are at a significantly higher risk of DVT due to immobilization and surgical trauma. Despite the widespread use of preventive measures, the incidence of perioperative DVT remains high, and more effective risk prediction tools are urgently needed to optimise clinical management (1, 2). Currently, researchers are focusing on routine haematological indicators such as red cell distribution width (RDW) as potential biomarkers to identify high-risk patient groups.

RDW is a routine blood test parameter that reflects anisocytosis, suggesting abnormal erythropoiesis or inflammation. Recent studies have found that elevated RDW is associated with the prognosis of various diseases, including venous thromboembolism (VTE) and myocardial infarction (3–5). In patients with lower extremity fractures, RDW may serve as a potential risk predictor for perioperative DVT. For example, studies have shown a positive correlation between RDW and the incidence of preoperative DVT, and its predictive value has been demonstrated in hip fracture patients (6). In addition, the mechanism of RDW on DVT formation may involve iron deficiency and inflammatory processes, which affect erythrocyte morphology and coagulation (7). However, its association varies in different populations: although RDW is often significantly elevated in the group of patients with DVT, it has not been identified as an independent predictor in some studies (8).

There is a notable scarcity of literature examining the role of RDW in perioperative DVT specifically in fracture populations, with existing studies predominantly focusing on older patients (6, 9, 10). The present study focuses on patients aged 18–75 years with lower-limb fractures, a comparatively under-studied population that includes patients with high-energy trauma and clinically relevant perioperative thrombotic risk. Because the trauma mechanism, comorbidity profile, and perioperative pathway may differ from those in exclusively geriatric cohorts, evidence derived specifically from this age range may contribute to more individualized risk assessment. Our study therefore provides preliminary evidence regarding the association between routinely available RDW parameters and perioperative DVT in this population.

## Materials and methods

### Patients

The study protocol was approved by the Ethics Committee of Xi'an Honghui Hospital (No. 2021026), which also waived the requirement for individual informed consent due to the retrospective design. All patient data were anonymized and handled in accordance with the principles of the Declaration of Helsinki. All methods were performed according to relevant guidelines and regulations. We retrospectively analysed the medical records of patients with lower limb fractures admitted to the Department of Traumatology and Orthopaedics of our hospital from February 2021 to February 2023.

Inclusion criteria: (1) age ≥18 years and ≤75 years; (2) freshly closed lower limb fracture treated with incisional reduction and internal fixation; and (3) completion of the ultrasound examination of the veins of the lower limbs at the time of admission and during the perioperative period.

Exclusion criteria: (1) old, open fractures or pathological fractures; (2) hematological disorders such as sickle cell disease or thalassemia; (3) a history of VTE; (4) thrombophilia genetic disorders; (5) history of anticoagulant use prior to admission; and (6) Incomplete clinical data.

### Study design

Patients underwent blood sampling and routine examination to complete preoperative preparation, and DVT prophylaxis was initiated on admission. For patients without contraindications, low-molecular-weight heparin (LMWH; 3,800 IU/0.4 mL once daily) was administered subcutaneously according to the guideline in CHEST 2012.15 (11) for DVT prophylaxis. In addition, we used a mechanical pressure pump (20 min, twice per day) to promote blood reflux. If DVT was diagnosed, it was managed by a vascular surgeon with a regimen that included subcutaneous injection of LMWH (3,800 IU/0.4 mL twice daily). If necessary, an inferior vena cava filter was placed preoperatively to prevent lethal pulmonary embolism. The primary indication was the preoperative diagnosis of a proximal (popliteal vein or above) or mixed (involving both proximal and distal segments) deep vein thrombosis (DVT), intending to proceed with urgent fracture fixation surgery to mitigate perioperative pulmonary embolism risk. The analyses in this study focused on the association between baseline parameters and subsequent DVT risk, based on data prior to DVT diagnosis and any resultant treatment escalation.

### Data collection

We collected clinical data through the hospital's surgical and anesthesia information system and electronic medical records. (1) Basic patient information including height, weight, body mass index (BMI), age, and gender. (2) Medical history including fracture site, preoperative smoking, alcohol consumption, hypertension, diabetes, coronary heart disease, cerebral infarction, arrhythmia, cancer, hepatitis, tourniquet, the time from injury to admission, time from injury to surgery and time of surgery. (3) Preoperative laboratory tests including Neutrophil count, Lymphocyte count, Hemoglobin (Hb), White blood cells (WBC), Platelet count (PLT), Red blood cell distribution width-standard deviation (RDW-SD), red cell distribution width-coefficient of variation (RDW-CV), Platelet distribution width (PDW), and ultrasound results of the veins of the lower limbs. All preoperative blood samples were collected within 24 h after admission and before the initiation of venous thromboembolism prophylaxis or surgical intervention.

All patients underwent venous ultrasound of both lower extremities using a standardized compression ultrasonography (CUS) protocol, examined by Philips cx50 GE Vivid E9 color Doppler ultrasound. All patients underwent the initial bilateral lower extremity venous ultrasound within 24 h of admission and again within 24 h preoperatively. The standardized CUS protocol included comprehensive assessment of the entire deep venous system of both lower extremities, specifically including the common femoral, femoral, and popliteal veins, as well as the calf veins (posterior tibial, peroneal, and muscular veins). Postoperative surveillance ultrasounds were performed on postoperative day 1 and again between 48 and 72 h post-surgery. Each patient was co-diagnosed by two experienced sonographers. Positive criteria for DVT were poor vascular compression, widening of venous internal diameters, intravascular filling defects, and absence of Doppler signals. Symptomatic DVT was defined as the presence of objective ultrasound-confirmed thrombosis accompanied by clinical signs such as unilateral limb swelling, pain, tenderness, or erythema. DVT detected by ultrasound surveillance in the absence of these clinical signs was classified as asymptomatic. Diagnosed DVT was further categorized according to anatomic location as distal (below the popliteal vein), proximal (popliteal vein and above) and mixed thrombus. Patients with proximal and mixed thrombi were implanted with an inferior vena cava filter before surgery.

### Statistical analysis

Given the retrospective design, an *a priori* power calculation was not conducted. However, the adequacy of the sample size for multivariable logistic regression was evaluated using the Events Per Variable (EPV) principle. With 234 DVT events and a maximum of 9 predictor variables entered into the models, the EPV ratio exceeded 26, which is well above the recommended threshold of EPV ≥10 for stable and reliable regression estimates. This confirms that the sample size was sufficient to support the multivariable analyses performed in this study.

Patients were divided into DVT and non-DVT groups according to lower-limb ultrasound findings. Statistical analyses were performed using SPSS 26.0 (SPSS Inc., Chicago, IL, USA). Continuous variables are presented as mean ± standard deviation and were compared using the independent-samples t test or Mann–Whitney U test, as appropriate. Categorical variables are presented as *n* (%) and were compared using the chi-square test or Fisher exact test. ROC curves were used to evaluate the discrimination of RDW-SD and RDW-CV. Cohort-specific exploratory cut-off values were selected by maximizing the Youden index, and the AUCs were compared using the DeLong test. Variables with *P* < 0.10 in univariate analyses were entered into multivariable logistic regression models. Multicollinearity was assessed using variance inflation factors, all of which were <2. Separate models were constructed for preoperative and new postoperative DVT, and a sensitivity analysis excluded patients with preoperative DVT. Time from injury to admission and time from injury to surgery were additionally included in sensitivity models. To address potential overfitting, internal validation using 1,000 bootstrap resamples was planned for both multivariable models; apparent AUC, optimism, optimism-corrected AUC, and optimism-corrected calibration slope were reported. Symptomatic and asymptomatic DVT cases were compared, and a parsimonious sensitivity logistic regression analysis compared symptomatic DVT with no DVT. Platelet count was entered as a continuous variable after division by 10; therefore, the reported OR represents the change in odds associated with each 10 × 10⁹/L increase in PLT. This scaling preserves the continuous information while providing a clinically interpretable effect estimate. Two-sided *P* < 0.05 was considered statistically significant.

## Results

### The characteristics of the included patients

A total of 766 patients were included in this study, with a mean age of 50.34 ± 15.13 years (range, 18–75 years), and 53.92% (413/766) were male. Baseline characteristics are shown in Table 1. Overall, 199 (25.98%) patients had ankle or foot fractures, 209 (27.28%) had tibia or fibula fractures, 115 (15.01%) had knee or peri-articular fractures, and 243 (31.72%) had hip or femur fractures. Perioperative DVT was confirmed by lower-extremity venous ultrasonography in 234 patients (30.55%): 175 (74.79%) had distal DVT, 33 (14.10%) had proximal DVT, and 26 (11.11%) had mixed DVT. Among the DVT events, 65 (27.78%) were symptomatic and 169 (72.22%) were asymptomatic. Seventy-eight events (33.33%) were detected preoperatively, whereas 156 (66.67%) were newly diagnosed postoperatively. No symptomatic pulmonary embolism occurred during the index hospitalization. Univariate comparisons showed significant between-group differences in age, fracture site, diabetes, Hb <120 g/L, PLT, RDW-SD, RDW-CV, and PDW (all *P* < 0.05).

**Table 1:** Comparison of baseline data between patients with DVT and non-DVT groups (*χ*±s)[*n* (%)].

Variable	DVT group (*n* = 234)	non-DVT group (*n* = 532)	*OR*(95%CI)	*p* value
Age (year)	57.11 ± 15.35	46.84 ± 14.70	1.047（1.035−1.059）	**<0**.**001**
Male (%)	131 (55.98)	282 (53.01)	1.128（0.828−1.536）	0.479
BMI ≥ 24 (%)	119 (50.85)	260 (48.87)	1.083（0.796−1.472）	0.613
Fracture site				**0**.**037**
Ankle/foot	45 (19.23)	154 (28.95)	1	0.022
Tibia/fibula	68 (29.06)	141 (26.50)	1.641 (1.061–2.562)	0.026
Knee/peri-articular	42 (17.94)	73 (13.72)	1.972 (1.192–3.261)	0.008
Hip/femur	79 (33.76)	164 (30.82)	1.651 (1.083–2.531)	0.022
Smoking (%)	81 (34.61)	159 (29.89)	1.242（0.895−1.723）	0.194
Drinking (%)	53 (22.65)	111 (20.86)	1.111（0.767−1.609）	0.579
Hypertension (%)	91 (38.89)	191 (35.90)	1.136（0.828−1.560）	0.430
Diabetes (%)	44 (18.80)	70 (13.16)	1.528（1.011−2.310）	**0**.**043**
Coronary heart disease (%)	38 (16.24)	61 (11.47)	1.497（0.966−2.320）	0.070
Cerebral infarction (%)	37 (15.81)	69 (12.97)	1.260（0.818−1.943）	0.294
Arrhythmia (%)	28 (11.97)	79 (14.85)	0.779（0.491−1.236）	0.289
Cancer (%)	32 (13.68)	49 (9.21)	1.562（0.971−2.510）	0.064
Hepatitis (%)	39 (16.67)	64 (13.68)	1.463（0.950−2.252）	0.083
Tourniquet (%)	179 (76.50)	382 (71.80)	1.278（0.895−1.825）	0.177
Time from injury to admission (days)				0.421
<1	148 (63.2%)	352 (66.2%)	1	
1–3	50 (21.4%)	106 (19.9%)	1.12 (0.76–1.65)	0.561
≥3	36 (15.4%)	74 (13.9%)	1.16 (0.75–1.79)	0.512
Time from injury to surgery (days)				
<2	30 (12.8%)	80 (15.0%)	1	0.372
2–4	110 (47.0%)	260 (48.9%)	1.13 (0.71–1.80)	0.601
≥4	94 (40.2%)	192 (36.1%)	1.31 (0.81–2.11)	0.272
Duration of operation (min)	89.25 ± 21.23	87.34 ± 18.46	1.502（0.231−2.284）	0.209
Neutrophil count (×10^9^/L)	6.65 ± 2.14	6.39 ± 1.98	1.646（0.622−2.861）	0.103
Lymphocyte count (×10^9^/L)	1.25 ± 0.42	1.28 ± 0.29	0.691（0.297−1.753）	0.254
Hb < 120 g/L (%)	103 (44.02)	165 (31.02)	1.749（1.274−2.401）	**<0**.**001**
WBC (×10^9^/L)	7.91 ± 2.13	7.66 ± 1.93	1.582（0.895−1.915）	0.110
PLT (×10^9^/L)	187.40 ± 36.15	168.29 ± 27.49	1.063 (1.027–1.235)	**0**.**022**
RDW-SD (fL)	45.97 ± 4.53	42.54 ± 2.37	1.495（1.388−1.610）	**<0**.**001**
RDW-CV (%)	13.47 ± 1.27	12.56 ± 0.68	1.229（1.134−1.566）	**<0**.**001**
PDW (fL)	13.07 ± 2.83	12.63 ± 2.16	1.113（1.071−1.358）	**0**.**019**
RDW-SD ≥ 43.9 fL (%)	122 (52.14)	168 (31.58)	2.360（1.722−3.234）	**<0**.**001**
RDW-CV ≥ 13.2% (%)	105 (44.87)	125 (23.50)	1.901（1.636−2.222）	**<0**.**001**

*P* values were calculated by the chi-square test, Fisher's exact test, Mann–Whitney U test, or t test. DVT, deep vein thrombosis; BMI, body mass index; Hb, hemoglobin; WBC, white blood cell; PLT, platelet count; RDW-SD, red cell distribution width-standard deviation; RDW-CV, red cell distribution width-coefficient of variation; PDW, platelet distribution width. Bold indicates *P* values <0.05. For PLT, the univariable OR corresponds to each 10 × 10⁹/L increase.

### Predictive value of RDW for perioperative DVT

**Figure 1:**
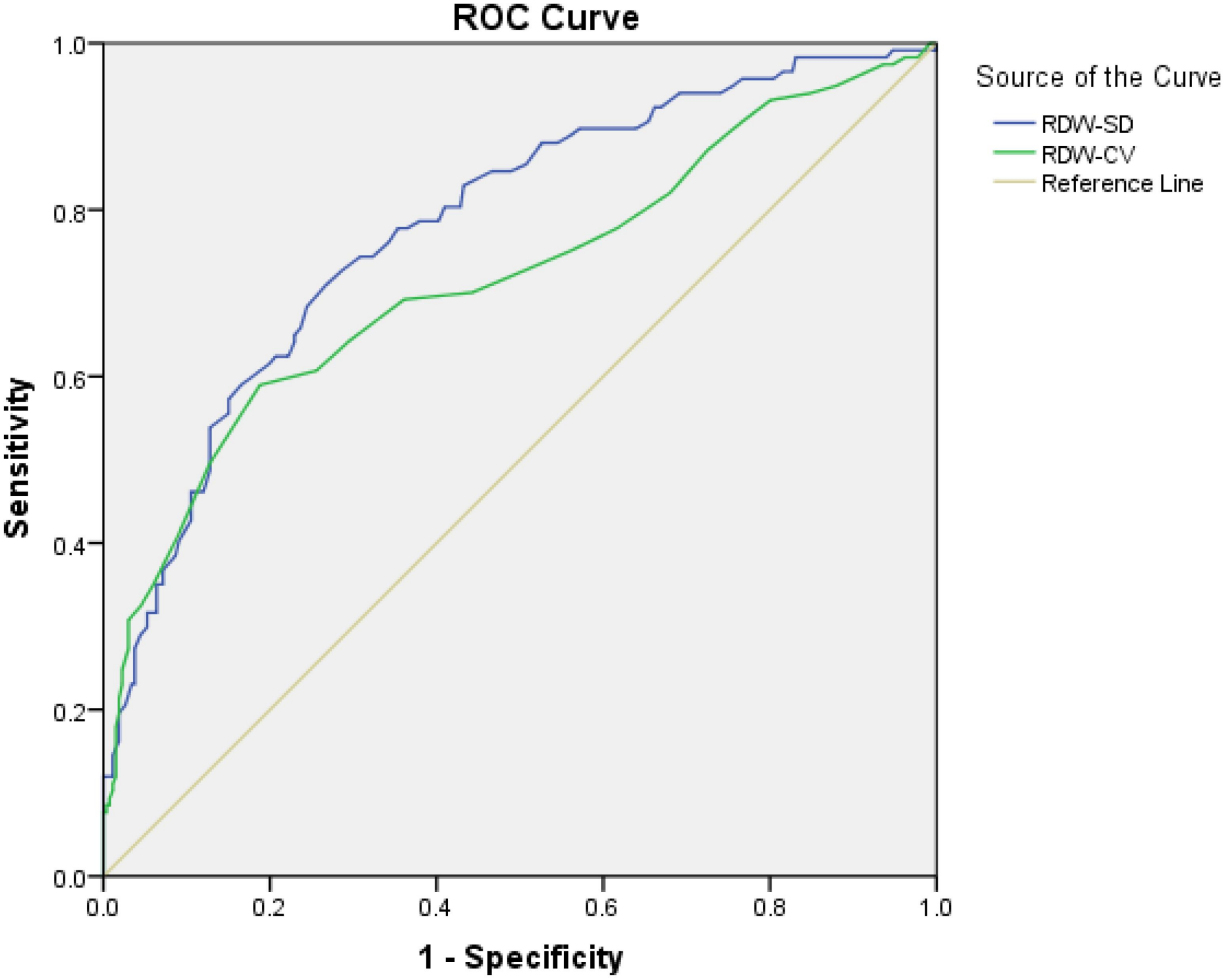
The predictive value of preoperative RDW-SD and RDW-CV for DVT in patients with lower limb fractures by ROC curve analysis. The AUC of RDW-SD for DVT was 0.781, with the cut-off value of 43.9 fL, a sensitivity of 70.94% and a specificity of 73.30%, respectively (95% CI: 0.745−0.817, *P* < 0.001). The AUC of RDW-CV for DVT was 0.719, with the cut-off value of 13.2%, a sensitivity of 58.97% and a specificity of 81.20%, respectively (95% CI: 0.676−0.761, *P* < 0.001).

ROC curve analysis (Figure 1) showed that RDW-SD discriminated perioperative DVT with an AUC of 0.781 [95% confidence interval (CI), 0.745–0.817; *P* < 0.001]. The cohort-specific exploratory cut-off value was 43.9 fL, with a sensitivity of 70.94% and a specificity of 73.30%. The AUC of RDW-CV was 0.719 (95% CI, 0.676–0.761; *P* < 0.001), with an exploratory cut-off value of 13.2%, a sensitivity of 58.97%, and a specificity of 81.20%. The DeLong test indicated better discrimination for RDW-SD than for RDW-CV (Z = 2.541, *P* < 0.05). The proportions of patients with RDW-SD ≥43.9 fL and RDW-CV ≥13.2% were higher in the DVT group than in the non-DVT group (both *P* < 0.001). In the RDW-SD stratified analysis (Table 2), the RDW-SD ≥43.9 fL subgroup had a higher mean age and higher proportions of DVT, diabetes, cancer, and Hb <120 g/L (all *P* < 0.05).

**Table 2:** Clinical factors in patients with low and high RDW-SD(*χ*±s)[n (%)].

Variable	RDW-SD		*p* value
	≥43.9(*n* = 290)	＜43.9(*n* = 476)	
Age	54.84 ± 14.14	47.01 ± 15.76	**<0**.**001**
Male (%)	165 (56.90)	248 (52.10)	0.197
BMI≥24 (%)	153 (52.76)	227 (47.69)	0.174
Fracture site			**0**.**018**
Ankle/foot	64 (22.1%)	135 (28.4%)	
Tibia/fibula	75 (25.9%)	134 (28.2%)	
Knee/peri-articular	43 (14.8%)	72 (15.1%)	
Hip/femur	108 (37.2%)	135 (28.4%)	
Smoking (%)	101 (34.83)	139 (29.20)	0.103
Drinking (%)	68 (23.45)	96 (20.17)	0.283
Hypertension (%)	113 (38.97)	169 (35.50)	0.335
Diabetes (%)	56 (19.31)	58 (12.18)	**0**.**007**
Coronary heart disease (%)	44 (15.17)	55 (11.55)	0.147
Cerebral infarction (%)	47 (16.21)	59 (12.40)	0.138
Arrhythmia (%)	45 (15.52)	62 (13.03)	0.335
Cancer (%)	41 (14.14)	40 (8.40)	**0**.**012**
Hepatitis (%)	47 (16.21)	56 (11.76)	0.080
Tourniquet (%)	222 (76.55)	339 (71.22)	0.106
Time from injury to admission (days)			0.652
<1	186 (64.1%)	314 (66.0%)	
1–3	60 (20.7%)	96 (20.2%)	
≥3	44 (15.2%)	66 (13.8%)	
Time from injury to surgery (days)			0.481
<2	38 (13.1%)	72 (15.1%)	
2–4	135 (46.6%)	235 (49.4%)	
≥4	117 (40.3%)	169 (35.5%)	
Duration of operation (min)	90.12 ± 16.53	88.31 ± 14.71	0.116
Neutrophil count (×10^9^/L)	6.71 ± 1.89	6.51 ± 1.62	0.120
Lymphocyte count (×10^9^/L)	1.21 ± 0.22	1.23 ± 0.19	0.184
Hb < 120 g/L (%)	125 (43.10)	143 (30.04)	**<0**.**001**
WBC (×10^9^/L)	7.75 ± 2.13	7.50 ± 1.98	0.100
PLT (×10^9^/L)	184.31 ± 37.25	179.79 ± 33.21	0.089
PDW (fL)	13.12 ± 2.96	12.88 ± 3.44	0.565
DVT (%)	113 (38.97)	121 (25.42)	**<0**.**001**

*P* values were calculated by the Chi-square test, Fisher's exact test, Mann–Whitney U-test, or t-test. DVT, deep vein thrombosis; BMI, body mass index; Hb, hemoglobin; WBC, white blood cell; PLT, platelet; RDW-SD, red cell distribution width-standard deviation; RDW-CV, red cell distribution width-coefficient of variation; PDW, Platelet Distribution Width. Bold indicates *P*-values less than 0.05.

### Univariate and multivariate logistic regression analyses of risk factors for DVT

Univariate logistic regression analysis (Table 1) showed that age, fracture site, diabetes, Hb <120 g/L, PLT, RDW-SD, RDW-CV, and PDW were associated with perioperative DVT (*P* < 0.05). Variables with *P* < 0.10 in the univariate analysis—age, fracture site, diabetes, coronary heart disease, cancer, hepatitis, Hb <120 g/L, PLT, PDW, and either RDW-SD ≥43.9 fL or RDW-CV ≥13.2%—were entered into the corresponding multivariable logistic regression models (Table 3).

**Table 3:** Multivariable analysis of factors associated with perioperative deep vein thrombosis.

Variable	OR	95%CI	*p* value*
Model 1 (RDW-SD)			
Age (per 1-year increase)	1.054	1.026−1.083	**<0**.**001**
PLT (per 10 × 10⁹/L increase)	1.115	1.076–1.155	**0**.**010**
Hb < 120 g/L	1.785	1.503−2.120	**<0**.**001**
RDW-SD ≥ 43.9 fL	3.372	2.335−4.765	**<0**.**001**
Model 2 (RDW-CV)			
Age (per 1-year increase)	1.035	1.017−1.054	**<0**.**001**
PLT (per 10 × 10⁹/L increase)	1.101	1.055–1.149	**0**.**008**
Hb < 120 g/L	1.539	1.365−1.735	**<0**.**001**
RDW-CV ≥ 13.2%	2.556	1.733−3.770	**<0**.**001**

DVT, deep vein thrombosis; PLT, platelet count; Hb, hemoglobin; RDW-SD, red cell distribution width-standard deviation; RDW-CV, red cell distribution width-coefficient of variation. Model 1 was adjusted for age, fracture site, diabetes, coronary heart disease, cancer, hepatitis, Hb <120 g/L, PLT, PDW, and RDW-SD ≥43.9 fL. Model 2 included the same covariates, replacing RDW-SD with RDW-CV ≥13.2%. PLT was divided by 10 before model fitting; thus, its OR is reported per 10 × 10⁹/L increase. Rescaling changes the OR and confidence interval but not the *P* value.

* *P* values were calculated using multivariable logistic regression. Bold values indicate *P* < 0.05.

Model 1 (including RDW-SD): after adjustment for age, fracture site, diabetes, coronary heart disease, cancer, hepatitis, Hb <120 g/L, PLT, and PDW, age (aOR, 1.054; 95% CI, 1.026–1.083; *P* < 0.001), higher PLT (per 10 × 10⁹/L increase: aOR, 1.115; 95% CI, 1.076–1.155; *P* = 0.010), Hb <120 g/L (aOR, 1.785; 95% CI, 1.503–2.120; *P* < 0.001), and RDW-SD ≥43.9 fL (aOR, 3.372; 95% CI, 2.335–4.765; *P* < 0.001) were independently associated with perioperative DVT.

Model 2 (including RDW-CV): after adjustment for the same covariates, age (aOR, 1.035; 95% CI, 1.017–1.054; *P* < 0.001), higher PLT (per 10 × 10⁹/L increase: aOR, 1.101; 95% CI, 1.055–1.149; *P* = 0.008), Hb <120 g/L (aOR, 1.539; 95% CI, 1.365–1.735; *P* < 0.001), and RDW-CV ≥13.2% (aOR, 2.556; 95% CI, 1.733–3.770; *P* < 0.001) were independently associated with perioperative DVT.

Sensitivity analyses additionally adjusting for time from injury to admission and time from injury to surgery showed that RDW-SD ≥43.9 fL (aOR, 3.351; 95% CI, 2.301–4.882; *P* < 0.001) and RDW-CV ≥13.2% (aOR, 2.542; 95% CI, 1.723–3.751; *P* < 0.001) remained significantly associated with DVT, whereas neither time variable was independently associated with DVT (both *P* > 0.05).

In the sensitivity analysis excluding the 78 patients with preoperative DVT, baseline RDW-SD ≥43.9 fL remained independently associated with new postoperative DVT (aOR, 2.921; 95% CI, 1.852–4.607; *P* < 0.001). Separate models also showed associations with both preoperative DVT (aOR, 3.182; 95% CI, 2.004–5.053; *P* < 0.001) and new postoperative DVT (aOR, 2.874; 95% CI, 1.823–4.530; *P* < 0.001).

### Additional internal-validation and symptomatic-DVT analyses

Bootstrap internal validation showed limited optimism. The RDW-SD model had an apparent AUC of 0.824, an optimism-corrected AUC of 0.811, and an optimism-corrected calibration slope of 0.94. The RDW-CV model had an apparent AUC of 0.795, an optimism-corrected AUC of 0.780, and an optimism-corrected calibration slope of 0.92.

Compared with asymptomatic DVT, symptomatic DVT showed a substantially higher proportion of proximal or mixed thrombosis, whereas differences in age, Hb <120 g/L, PLT, RDW-SD, and RDW-CV were modest and did not reach statistical significance in the illustrative analysis (Supplementary Tables S1).

In a parsimonious sensitivity model comparing symptomatic DVT with no DVT, RDW-SD ≥43.9 fL remained associated with symptomatic DVT (aOR, 2.28; 95% CI, 1.29–4.03; *P* = 0.005), together with age and PLT (Supplementary Tables S2).

## Discussion

To our knowledge, this is the first large-scale study to focus on patients under 75 years old with lower limb fractures and systematically evaluate the predictive value of red cell distribution width (RDW) for perioperative deep vein thrombosis (DVT). This study confirmed that red cell distribution width standard deviation (RDW-SD) and coefficient of variation (RDW-CV) were independent predictors for the occurrence of DVT through a retrospective analysis of 766 patients. Multivariate logistic regression analysis showed that RDW-SD ≥43.9 fL and RDW-CV ≥13.2% increased the risk of DVT by 3.372-fold and 2.556-fold, respectively. In addition, the predictive efficacy of RDW-SD (AUC=0.781) was significantly better than that of RDW-CV (AUC=0.719) (Z = 2.541, *P*＜ 0.05). Advanced age, high platelet count (PLT), and Hb＜ 120 g/L were also identified as independent risk factors for DVT. Collectively, these findings offer novel perspectives for early risk stratification and personalized prophylaxis in this previously underexplored surgical population.

### Evidence supporting the correlation between RDW and DVT risk

The results of this study are consistent with the existing consensus in the literature that elevated RDW is associated with an increased risk of DVT. RDW, as a reflection of erythrocyte heterogeneity, has been identified as an independent risk factor for VTE in several studies. For example, Xiong et al. (12) found a positive association between elevated RDW and preoperative DVT in patients undergoing total joint arthroplasty (TJA) (*P* < 0.05), and the association was independent of other haematological parameters. Febra et al. (13) showed that elevated RDW values have a good predictive value for preoperative DVT (AUC = 0.685), suggesting its potential in prospective screening. Cheng et al. (6) also found a notable predictive ability of RDW for DVT (AUC = 0.532) in their study of hip fracture in the elderly. Whereas the predictive efficacy of RDW-SD (AUC = 0.781) and RDW-CV (AUC = 0.719) was observed to be higher than that of previous studies in this study, we considered its better predictive potential in younger (< 75 years old) lower extremity fractures, and we confirmed its independence by multivariate analyses, which further extends the applicability of the RDW in different fracture populations.

Furthermore, fracture site is a well-established factor influencing DVT risk. In our univariate analysis, the distribution of fracture sites differed between the DVT and non-DVT groups (*P* = 0.037), with hip/femur fractures being more prevalent among patients with thrombosis. However, fracture site was not independently associated with DVT after multivariable adjustment. This finding indicates that the relationship between fracture location and thrombosis may overlap with age, injury characteristics, immobilization, and hematologic abnormalities. It does not exclude fracture severity as a clinically important factor, and RDW should not be interpreted as a direct mechanistic marker in place of these established determinants.

Different forms of RDW may have different discriminatory performance. In this study, RDW-SD showed better discrimination than RDW-CV (*P* < 0.05). Similar differences between RDW-SD and RDW-CV have been reported in other clinical settings (14–17). RDW-SD directly describes the width of the erythrocyte volume distribution and may be less dependent on the mean corpuscular volume than RDW-CV. Nevertheless, the present finding should be interpreted as comparative performance within this cohort, and RDW-SD should be considered a candidate adjunctive marker rather than a stand-alone screening test. External validation is required before selecting either parameter for routine thrombotic risk assessment.

The multivariable models integrated RDW with age, Hb <120 g/L, PLT, and other measured clinical factors. The associations of advanced age and low hemoglobin with DVT are consistent with previous studies in fracture and arthroplasty populations (3, 12). These results extend the available evidence to patients aged 18–75 years, while the absence of several inflammatory, iron-metabolism, and coagulation markers limits mechanistic interpretation.

### Exploration of the potential biological mechanisms

The observed association between elevated RDW and perioperative DVT may reflect several overlapping pathways, including erythrocyte heterogeneity, anemia, inflammation, iron metabolism, and the systemic response to trauma. Because the present study was observational and did not include several key mechanistic biomarkers, the following explanations should be regarded as biologically plausible hypotheses rather than demonstrated causal pathways.

First, elevated RDW reflects anisocytosis and may accompany iron deficiency, chronic disease, nutritional deficiency, or ineffective erythropoiesis. Patients with lower-limb fractures may present with pre-existing anemia, trauma-related blood loss before admission, or chronic comorbid conditions affecting erythropoiesis. In this study, the RDW-SD ≥43.9 fL group had a higher proportion of Hb <120 g/L. However, hemoglobin alone cannot establish iron deficiency, and serum ferritin, transferrin saturation, and hepcidin were unavailable. Therefore, the observed relationship may partly reflect an underlying anemia or iron-metabolism phenotype. Erythrocyte heterogeneity may also be associated with reduced deformability and altered microcirculatory flow, but this mechanism was not directly evaluated.

Second, inflammation may contribute to both elevated RDW and thrombosis. Trauma and surgical stress can promote inflammatory signaling and coagulation activation (18). In the present data, Neutrophil count and PDW were higher in the DVT group in this study, which is consistent with the theory of prothrombotic inflammatory state. Chronic inflammation also inhibits the erythropoietin response, leading to the release of immature erythrocytes into the bloodstream, which further pushes up RDW values. A prospective study by Ellingsen et al. (3) found that RDW was an independent predictor of VTE after myocardial infarction and stroke, and that its effect was partially mediated by inflammatory mediators (*P* < 0.01). In the present study, diabetes was used as a covariate in a multivariate model (*P* < 0.05), and its pro-inflammatory status may underlie the association. In addition, history of cancer and hepatitis were included as covariates in the model and the proportion of cancer was also higher in the subgroup of RDW threshold (*P* < 0.05), suggesting that the complex mechanisms of cancer and chronic inflammation may favour the amplification of the RDW effect, which is also in line with the observation of Ellingsen et al. (3).

### Clinical significance and optimisation of risk screening strategies

The present findings suggest that elevated RDW-SD and RDW-CV are independently associated with perioperative DVT and may contribute to risk stratification when interpreted together with established clinical factors.

Firstly, RDW, as a routine blood test, has the advantage of being low-cost and easily accessible, and can be conveniently integrated into the DVT screening process, especially for resource-limited settings (19, 20). The high AUC value (0.781) of RDW-SD and its good performance (sensitivity 70.94%, specificity 73.30%) with a critical value (43.9 fL) in the present study support its value in routine preoperative assessment of fracture patients under 75 years of age.

Second, combining RDW with other indicators (such as PLT, Hb, and age) to construct a prediction model can significantly improve the accuracy of risk stratification. The multifactor model in this study integrated these key factors, similar to the strategy of Zeng et al. (21). The finding of high PLT as an independent risk factor in the present study is consistent with the findings of Malte et al. (22) on platelet parameters and risk of cancer-related thrombosis. The inclusion of Hb＜ 120 g/L, on the other hand, highlights the importance of perioperative anaemia management in reducing the risk of DVT. Future clinical guidelines may consider the inclusion of RDW indicators and refer to the nomogram-based approach proposed by Zheng et al. (19) to develop individualised prophylaxis protocols (such as pharmacological prophylaxis intensity, mechanical prophylaxis duration, and frequency of monitoring) for patients with different risk classes.

The symptomatic-DVT sensitivity analysis provides an additional, clinically oriented perspective. After excluding asymptomatic DVT cases, RDW-SD ≥43.9 fL remained associated with symptomatic DVT (aOR, 2.28; 95% CI, 1.29-4.03; *P* = 0.005), whereas Hb <120 g/L did not remain statistically significant in this parsimonious model (aOR, 1.52; 95% CI, 0.89−2.60; *P* = 0.126). This pattern suggests that the RDW-SD association was not entirely dependent on anemia as defined by hemoglobin alone. However, because only 65 symptomatic events were available, this analysis should be interpreted as supportive rather than confirmatory.

In addition, this study reported for the first time a significantly higher proportion of younger fracture patients with a history of cancer (*P*＜ 0.05) who had a significantly elevated RDW (RDW-SD ≥43.9 fL). This finding has a dual significance: on the one hand, cancer itself directly increases the risk of DVT through the release of procoagulant substances (such as tissue factor, cancer procoagulant); on the other hand, tumour-associated anaemia and myelosuppression by chemotherapeutic agents can lead to elevated RDW (23, 24). Therefore, in young fracture patients with significantly elevated RDW, clinicians should be alert to the possibility of potentially occult malignancy and recommend targeted tumour marker screening when conditions permit.

In conclusion, this study provides unique insights into the perioperative management of DVT in patients under 75 years of age with lower extremity fractures. Traditionally, DVT risk studies have focused on older patients (> 75 years of age), and younger patients are easily overlooked. Although the proportion of comorbidities (such as diabetes, cancer) may be lower in this group than in the older age group, the incidence of perioperative DVT is still high, and the incidence of DVT in the present study could be up to 30.55%, which is similar to the incidence in the study by Cheng Ren et al. (31.30%) (25, 26), underscoring the need for tailored risk assessment and prevention in this population. We consider that firstly, the endothelial damage is more severe in this population because of the high energy of trauma due to their high mobility; secondly, there is insufficient clinical awareness of the risk of DVT in this patient population and inadequate application of prophylactic measures; and lastly, physiological compensations in this age group may mask early symptoms and lead to a delay in diagnosis. The results strongly challenge the traditional concept that DVT is an exclusive complication in elderly fracture patients and sound an alarm for clinical practice. We argue that the practical strength of RDW lies in its accessibility and low cost as a routine parameter. It can be seamlessly integrated into preoperative assessments, providing an immediate, objective data point for enhanced risk stratification, particularly valuable in resource-conscious settings. Therefore, the primary clinical utility of RDW in this context is to serve as an early, routine, and inexpensive “red flag” for personalized risk stratification. For a patient under 75 years with a lower limb fracture, an RDW-SD value ≥ 43.9 fL should prompt heightened clinical vigilance, potentially refining prophylactic decisions independently of or in conjunction with traditional clinical scores. Practical considerations might include scheduling earlier or more frequent ultrasound surveillance, reviewing current prophylaxis protocols, and enhancing patient and caregiver education regarding DVT symptoms. However, decisions regarding changes to anticoagulation intensity should not be based on RDW alone and require validation in prospective interventional studies.

### Limitations and outlook

This study has several limitations. First, its retrospective, single-center design limits causal inference and external generalizability. Although Hb and several clinical comorbidities were included in the adjusted models, ferritin, C-reactive protein, fibrinogen, D-dimer, and other iron-metabolism, inflammatory, and coagulation markers were not routinely available. Residual confounding therefore cannot be excluded, and RDW should be interpreted as an associated risk marker rather than a mechanism-specific or stand-alone predictor. Second, the RDW thresholds identified in this cohort require prospective multicenter external validation and may vary according to patient characteristics and laboratory platforms. Third, most DVT events were asymptomatic and identified through protocolized ultrasonography; analyses restricted to symptomatic DVT contained fewer events and should be interpreted cautiously. Fourth, although time from injury to admission and time from injury to surgery were included in sensitivity analyses, their retrospective recording and categorical modeling may not fully capture trauma severity, referral pathways, or perioperative delay. Fifth, interobserver agreement for ultrasound diagnosis and post-discharge thrombotic outcomes were not assessed. Finally, internal bootstrap validation can quantify optimism within the present dataset but cannot replace independent external validation. Future prospective multicenter studies should incorporate iron-metabolism, inflammatory, and coagulation biomarkers and evaluate clinically symptomatic and post-discharge thromboembolic outcomes.

## Conclusion

Elevated baseline RDW-SD and RDW-CV were independently associated with perioperative DVT in patients aged 18–75 years with lower-limb fractures. Meanwhile, advanced age, Hb＜120 g/L, and high platelet count (PLT) were also significantly associated with the risk of DVT. The predictive efficacy of RDW-SD was better than that of RDW-CV, and it can be used as a potential biomarker for perioperative DVT screening. This finding provides a new basis for individualised thromboprophylaxis strategies in young fracture patients, especially for early risk assessment in resource-limited scenarios.

## Data Availability

The raw data supporting the conclusions of this article will be made available by the authors, without undue reservation.
