# Supplementary material for: Correlation between red blood cell distribution width and deep vein thrombosis in patients with lower limb fractures
Source: Front Cardiovasc Med. 2026 Jul 31;13:1841161. doi: 10.3389/fcvm.2026.1841161 (PMC13472994; doi:10.3389/fcvm.2026.1841161)
Supplement: Supplementary file 1 [file Datasheet1.pdf]

Supplementary Tables S1. Comparison between symptomatic and asymptomatic DVT

**Variable**	**Symptomatic DVT (n=65)**	**Asymptomatic DVT (n=169)**	**P value**
Age, years	59.2 ± 14.5	56.3 ± 15.5	0.181
Male, n (%)	39 (60.0)	92 (54.4)	0.443
Hb <120 g/L, n (%)	34 (52.3)	69 (40.8)	0.113
PLT $\times 10^9$/L	194.2 ± 39.0	184.8 ± 34.5	0.091
RDW-SD, fL	46.6 ± 4.8	45.7 ± 4.4	0.192
RDW-SD ≥43.9 fL, n (%)	39 (60.0)	83 (49.1)	0.135
RDW-CV, %	13.72 ± 1.35	13.37 ± 1.22	0.071
RDW-CV ≥13.2%, n (%)	35 (53.8)	70 (41.4)	0.087
DVT location, n (%)			**<0.001**
Distal	27 (41.5)	148 (87.6)	
Proximal	20 (30.8)	13 (7.7)	
Mixed	18 (27.7)	8 (4.7)	

DVT: deep vein thrombosis; Hb: hemoglobin; PLT: platelet; RDW-SD: red cell distribution width-standard deviation; RDW-CV: red cell distribution width-coefficient of variation; Bold indicates *P*-values less than 0.05.

Supplementary Tables S2. Sensitivity logistic regression for symptomatic DVT versus no DVT

**Predictor**	**aOR**	**95% CI**	**P value**
Age (per 1-year increase)	1.038	1.018–1.059	**<0.001**

Supplementary Tables S2. Sensitivity logistic regression for symptomatic DVT versus no DVT

**Predictor**	**aOR**	**95% CI**	**P value**
PLT (per 10×10^9/L increase)	1.071	1.016–1.130	**0.011**
Hb <120 g/L	1.52	0.89–2.60	0.126
RDW-SD ≥43.9 fL	2.28	1.29–4.03	**0.005**

PLT: platelet. Hb: hemoglobin. RDW-SD: red cell distribution width-standard deviation. The illustrative model includes four predictors to limit overfitting with 65 symptomatic events. Asymptomatic DVT cases should be excluded from the non-event group. PLT is expressed per 10×10^9/L increase, consistent with the scaling used in the primary models. Bold indicates *P*-values less than 0.05.
